# Quality of life and social contacts in old age

**DOI:** 10.1093/eurpub/ckac130.112

**Published:** 2022-10-25

**Authors:** K Loeffler, L Weidinger, M Bohnstingl, C Pammer, J Goldgruber

**Affiliations:** 1 Albert Schweitzer Institute for Geriatric Medicine, Geriatric Health Care Centers, Graz, Austria; 2 Sozialarbeit, Medius Zentrum für Gesundheit, Graz, Austria

## Abstract

**Background:**

Changes in lifestyle, housing and social relationships have a verifiable impact on aging. Older adults of today have different ideas about housing and residential mobility than previous generations. Consequently, the importance of innovative types of housing for senior citizens, which allow largely independent housekeeping and provide integration into preexisting social structures, is increasingly in the focus of public health debates.

**Methods:**

Face-to-face interviews with 32 tenants of Wohnoase Robert Stolz in Graz were conducted between July 2018 and December 2020. The subjects were interviewed twice (T1: at move-in, T2: 6-12 months after moving-in). WHOQOL-BREF and WHOQOL-Old were used to assess health-related quality of life. Furthermore, the ego-centered social network map and a questionnaire to assess subjective sense of safety at home were applied.

**Results:**

Tenants’ health-related quality of life improved by an average of 11% after 6-12 months in the area of environmental conditions for health promotion ((T1: 3.83; T2: 4.25). Residential satisfaction (T1: 3.44; T2: 4.3; +25%) as well as subjective feeling of safety in their own homes (T1: 4.19; T2: 4.81; +14.8%) rose significantly. Tenants on average had about 6 more social contacts than before moving in (52.2%). In contrast, no significant changes were evident in tenants’ self-assessment of their physical, mental, and social health.

**Conclusions:**

Results indicate that moving into assisted living can lead to psychosocial stabilization and an increased sense of security. Over time, deteriorations in physical health can be observed, which can most likely be attributed to advanced ageing. Conducting an evaluation study with a higher number of participants and a control group is recommended.

**Key messages:**

- Moving into an assisted living facility led to psychological stabilization and a heightened sense of security among the participants.
- Further, the amount of social contacts increased over time.

